# The Influence of Body Weight on Chosen Physiological Parameters in Wrestling

**DOI:** 10.2478/hukin-2013-0032

**Published:** 2013-07-05

**Authors:** Hrvoje Karninčić, Saša Krstulović, Mario Baić

**Affiliations:** 1 Chair of theory and metodology of Wrestling, Faculty of Kinesiology, University of Split, Croatia.; 2 Chair of theory and metodology of Judo, Faculty of Kinesiology, University of Split, Croatia.; 3 Chair of theory and metodology of Wrestling, Faculty of Kinesiology, University of Zagreb, Croatia.

**Keywords:** combat sport, anaerobic, lactate, glucose, weight categories

## Abstract

In this study, the authors attempted to determine whether the dynamics of blood lactate and glucose in wrestling depend on the weight class. Blood lactate and glucose curves during and after a wrestling match were determined. We also explained the dynamics of blood lactate and glucose in the context of recent glucose and lactate metabolism research. A sample of 60 youth wrestlers (15–20 years) were divided into three weight groups. Each athlete participated in one wrestling match. During the fight, the athletes’ heart rate, glucose, and blood lactate were measured. The differences in body mass between the athletes did not affect the dynamics of lactate and glucose in wrestling competition (Fisher LSD test). We established that lactate and glucose dynamics are the same for all weight groups (Fisher LSD-Lactate 1 < 2 < 3 = 4 > 5, Fisher LSD-Glucose 1 = 2 < 3 < 4 < 5). Understanding lactate and glucose metabolism in wrestling is important for wrestling coaches because they need to evaluate a wrestler’s anaerobic energy status.

## Introduction

There are multiple forms of wrestling but there are only two styles of competitive Olympic wrestling: free-style and Greco-Roman style. In all styles of wrestling, the objective is to establish physical control over the opponent ( Horswill, 1992 ). A wrestling match is an intermittent physical exercise of variable intensity ( Hübner-Wozniak, 2006 ) and is characterised by sudden, explosive attacks and counterattacks that are executed repeatedly ( Hubner-Wozniak et al., 2004 ). In wrestling, as in many other sports, both anaerobic and aerobic energy systems are employed to various degrees. Anaerobic metabolism is predominantly used during the fight. Aerobic metabolism primarily contributes during recovery periods ( Hubner-Wozniak et al., 2004 ). From a metabolic perspective, the acid–base balance is severely disrupted. For example, a Greco-Roman or freestyle match lasts between 6 and 8 minutes (including overtime) and can elevate blood lactate concentrations in excess of 15 mmol/L, sometimes reaching nearly 20 mmol/L ( Kraemer et al., 2001 ). Physiological tests to evaluate anaerobic power and capacity in wrestling are based on the observation of certain metabolites in the blood, such as glucose or lactate. Lactate is the product of the anaerobic breakdown of glucose in tissues. While earlier research demonstrated that lactate was a waste product and a cause of acidosis, new findings have shown that lactate not only does not cause acidosis ( Robergs et al., 2004 ) but it is also a very useful carbohydrate in times of increased energy demand ( Miller et al., 2002 ). New theories have been unhelpful for developing sports diagnostics. Current research allows us to understand that lactate production during exercise acts as a physiological signal for the activation of a vast transcription network that affects monocarboxylate transporter protein expression and mitochondrial biogenesis, thereby explaining how training increases the capacity for lactate clearance via oxidation ( Hashimoto and Brooks, 2008 ). In spite of these issues, lactate concentration is considered a good indicator of training load. Lactate concentration correlates highly with the intensity of performance and can be regarded as an indicator of an optimal training stimulus ( Bourdon, 2000 ). There is no relationship between lactate concentration and success in wrestling ( Hubner-Wozniak et al., 2004 ). The winner in a wrestling match exerts more effort; however, better technical level conserves his energy. Some hypotheses are still acceptable, but lactate research in wrestling should be revised because of the new discoveries in lactate metabolism and periodic rule changes in wrestling. Lactate production can be stimulated by catecholamines. During stress, adrenaline can activate glycolysis in muscle tissue. A higher rate of glycolysis and increased lactate concentration are independent of tissue hypoxia. A review of the literature showed that weight differences within this sample (high level athletes with 4–6 years of competition experience) should not cause differences in adrenaline concentration. Therefore, it should not be taken under consideration. Since wrestling is a sport with a high energy demand in a relatively short period, it causes a large increase in glucose concentration ( Loupos, 2008 ). Several authors have connected lactate and glucose levels and applied glucose research to the diagnosis of anaerobic capacity ( Simoes et al., 2010 ; Sotero et al., 2009 ). Altogether, these findings suggest that measuring glucose concentration in wrestling for diagnostic purposes might be challenging.

Common anaerobic calculations use body weight in the formulas to calculate final results, but this is not a general practice for measuring lactate. The question is if two wrestlers of significantly different body mass and the same values of blood lactates after bout, have the same anaerobic capacity. The use of weight classes in wrestling is based on the assumption that differences in body weight can create an advantage for the larger wrestler. Therefore, the establishment of weight classes is a rational solution to the inequity created by size differences. The international wrestling rules (FILA) define 10 weight categories for cadets and 8 weight categories for juniors. The weight difference between the lightest and heaviest members of a wrestling team is approximately 60 kg. Lightweight wrestlers have different relationships between standing and parterre wrestling, different technical and tactical structures, and a different number of points won during the round when compared to heavyweight wrestlers ( Dokmanac et al., 2011 ). These differences often mean that different energy pathways are used during the match. Body build and the composition of wrestlers depend on their weight category ( Sterkowicz-Przybycien et al., 2011 ). Adipose tissue significantly contributes to systemic lactate turnover ( Van-Hall, 2010 ). Taking into consideration that the style of wrestling match and body composition are not the same in different weight categories, physiological responses (lactate, glucose) to wrestling may be different between weight categories.

In the current study, we asked whether the physiological response to a wrestling match differs between particular weight categories. A sparring match between two equal rival wrestlers is structurally similar to a competition match and may be carried out under controlled conditions. In this study, the authors investigated the lactate and glucose dynamics during a Greco-roman wrestling match in three different weight classes.

The objective of this research was to determine whether there were significant differences in the measured concentrations of lactate and glucose before, during, and after a wrestling match between lightweight, middleweight, and heavyweight youth wrestlers.

## Material and Methods

### Subjects

The study was conducted with 60 youth wrestlers, 15–20 years old, who were junior and cadet (according to international wrestling rules) members from 13 Croatian wrestling clubs. Each of the subjects participated in the Croatian Greco-Roman wrestling championship for juniors or cadets and placed between the first and tenth place. Wrestlers that placed below the tenth position were not considered for this study because some of them were beginners and it was unclear whether we could measure the impact of wrestling training. Differences in anaerobic energy production from glycolysis occur in later years ( Korhonen et al., 2005 ). Therefore, it is reasonable to observe these age categories as a group. The sample was divided into three weight categories: lightweight (n = 20; 57 ± 6 kg), middleweight (n = 20; 70 ± 2 kg) and heavyweight (n = 20; 88 ± 13 kg). The study protocol was approved by the ethical committee of the Faculty of Kinesiology in Split (Croatia) and written informed consent to participate in the study was signed by each subject or his parents prior to commencement.

### Measures

Ten physiological variables for each weight category were measured:

1. Lactate concentration before the match—after the warm-up,
2. Lactate concentration after the first bout,
3. Lactate concentration after the second bout,
4. Lactate concentration after the third bout,
5. Lactate concentration in the 5th min of recovery,
6. Glucose concentration before the match—after the warm-up,
7. Glucose concentration after the first bout,
8. Glucose concentration after the second bout,
9. Glucose concentration after the third bout,
10. Glucose concentration in the 5th min of recovery.

### Procedures

The concentration of lactate in blood was measured using the Accutrend lactate device; the validity was established by Baldari ( Baldari et al., 2009 ). The amount of glucose in blood was determined using an Accu-Chek Active device, and validity was established by Freckmann ( Freckmann et al., 2010 ). Heart rate was measured using the Polar PE3000 Heart Rate Monitor (Polar Electro Oy, Kempele, Finland). For the purpose of calculating body mass index, the subjects’ body mass and height were measured. Body mass was measured with a medical scale and a Martin’s anthropometer was used for measuring body height.

Subjects were instructed to follow a normal lifestyle by maintaining daily habits and avoiding any medication, alcohol, and caffeine as well as vigorous exercise within 24 hours of the test. Testing started at 10:00 AM. The warm-up, which was 15 min in duration, consisted of general preparatory exercises for 5 min, stretching exercises for 5 min, and specific individual and pair exercises for 5 min. After warming up, control matches were held according to the current international wrestling rules: three two-minute rounds (30 s rest periods between rounds). The only exception to the rules was the duration of the match, which can end before time expires due to a fall or technical superiority (6 points difference) during competition. However, for the purpose of this research all matches lasted until time expired. After a fall, wrestlers returned to a neutral position and continued to wrestle. To achieve similar fight intensity, each wrestler had an opponent in the same weight category (according the FILA wrestling rules), skill level, and training experience. To match pairs of rivals, an expert team was used (trainer of cadet national team, coach of junior national team and head coach of the Croatian Greco-roman national teams). The team of experts was present at the state championships for juniors and cadets, and they were familiar with the wrestlers who participated in this research. The sports medicine laboratory “Diomed” from Split, Croatia, was hired for the purposes of this research.

Blood samples were taken by medical laboratory technicians. Laboratory technicians took blood samples after the warm-up, after each round and after 5 min of recovery. Immediately after the bout a team of laboratory technicians approached the wrestler on the wrestling mat, recorded his heart rate, and took blood samples (capillary blood from a finger tip) during the 30 s between bouts. Both wrestlers took a 5 min rest period after the match before the last evaluation.

### Analysis

Statistical analysis was carried out using the statistical package Statistica version 7.0. (Statsoft, USA). All variables were analysed using descriptive statistics (mean, standard deviation, sample minimum, and sample maximum). Normal distribution of the data was confirmed through the Kolmogorov-Smirnov test. To determine the differences in lactate and glucose values between bouts and between weight groups, ANOVA was used for repeated measurements with the Fisher’s test in the post-hoc analysis. Pearson correlations were used to examine the relationship between blood lactate and glucose concentration during the fight, and p<0.05 was considered statistically significant.

## Results

Pre-match heart rate was elevated from baseline values because of the warm-up but it was comparable between the weight groups and varied from a mean of 104 bpm for lightweight to 107 bpm for middle and heavyweight wrestlers. After each bout the heart rate values confirmed the high intensity of the matches for lightweights (190; 189; 191 bpm), middleweights (184; 188; 189 bpm) and heavyweights (189; 191; 189 bpm). After 5 min of passive rest, the heart rates decreased to 120 bpm for the lightweight group, 123 bpm for the middleweight group, and 125 bpm for the heavyweight group. The warm-up slightly raised the blood lactate concentration from baseline values (1.5–2.0 mmol/L). For the lightweight group of wrestlers, the baseline value was 2.5 mmol/L; for the middleweight group, it was 3.1 mmol/L; and for the heavyweight group, it was 2.9 mmol/L. After the first bout, the lactate concentration rose in a statistically significant manner for all groups (5.3–6.7 mmol/L). The blood lactate concentration continued to rise after the second bout. This increase was much lower for all groups (1.7–2.2 mmol/L higher than the levels measured after the first bout) but it was still significant. The lowest increase in the blood lactate concentration was recorded after the third bout and was not statistically significant for all three groups (0.3–0.7 mmol/L higher than the levels measured after the second bout). After 5 min of sedentary rest, blood lactate concentration showed a statistically significant decrease for all groups (1.9–2.5 mmol/L lower than the levels measured after the fight).

After the warm-up, the glucose concentrations for all groups were near the upper level of the reference range. After the first bout, the rise in glucose concentration was not statistically significant in all three weight groups (02–0.6 mmol/L higher than that measured before the fight). The rise in glucose concentration was significant in all other measurements for all groups.

The correlation between blood lactate and glucose concentration decreased in the early stages of the match and increased at the end of the match. However, the increase was only significant after the third bout (r = 0.63) and after 5 minutes of rest (r = 0.46) for the lightweight group of wrestlers.

This study confirmed the same trend for lactate (Fisher LSD 1 < 2 < 3 = 4 > 5) and glucose (Fisher LSD 1 = 2 < 3 < 4 < 5) dynamics during Greco-roman wrestling matches for lightweight, middleweight, and heavyweight youth wrestlers.

## Discussion

Previous measurements of lactate concentration prior to a wrestling match showed concentrations between 1.7 and 2.2 mmol/L ( Kraemer et al., 2001 ) and between 1.3 and 2.5 mmol/L ( Barbas et al., 2011 ). These values were slightly higher than the values measured in this study. In those investigagions lactate concentration was measured before the warm-up. In the current study, the first measurement before the match was after a 15 min warm-up protocol, which took place under aerobic conditions and caused a slight elevation in blood lactate concentration compared with baseline values. The glucose concentrations before the wrestling match in previous studies were between 4.8 and 5.5 mmol/L ( Barbas et al., 2011 ; Kraemer et al., 2001 ) compared with 5.3–5.6 mmol/L in our research. It is obvious that the 15 min warm-up under aerobic conditions does not affect glucose concentration. The average heart rate of 188 bpm after each bout confirms the high intensity of the fight. After a wrestling match, other authors have measured similar values, from 175 to 188 bpm ( Barbas et al., 2011 ; Kraemer et al., 2001 ). Clearly, the heart rate values in these studies are similar. They confirm that the intensity of the wrestling matches were in the maximal load zone. Lactate and glucose are influenced by the intensity and duration of exercise ( Gladden, 2000 ). Therefore, it is logical that there is a correlation between them ( Sotero et al., 2009 ). The lack of a correlation in the current study can be explained by the distinct metabolic stress that occurs during a wrestling match. We assume that the relatively low correlation between lactate and glucose in such a situation occurs due to the adaptation of the organism. No differences were observed in the dynamics of lactate and glucose between wrestlers of different weight classes. Despite the differences in correlations between the substrates, the obtained data suggest that all of the weight classes can be considered as one group.

The most significant blood lactate increase was after the first bout. Wrestling bouts are short (2 min according to the last international wrestling rules), and the wrestlers must exert maximum effort from the first moments of the fight. The great amount of load in the second minute, under anaerobic conditions, leads to blood lactate over 8 mmol/L in seniors ( Karnincic et al., 2009 ). A similar result was obtained in young wrestlers (from 8.2 to 9.2 mmol/L). The increase in skeletal muscle glucose uptake during exercise results from a coordinated increase in the rate of glucose delivery (higher capillary perfusion), surface membrane glucose transport, and intracellular substrate flux through glycolysis ( Rose and Richter, 2005 ). The metabolic pathway consumed energy, although glucose uptake is slower. The increase is significant after the second bout (after approximately 5 minutes). The increase in glucose concentration is more significant at every subsequent measurement, contrary to the trend in lactate concentrations. The age-related differences in blood lactate concentrations during exercise are not significant before 60–70 years of age ( Korhonen et al., 2005 ). Lactate concentration after the second bout in young wrestlers was similar to that in seniors. Senior blood lactate concentrations were between 11 and 13 mmol/L ( Karnincic et al., 2009 ) and between 10 and 11 mmol/L in young wrestlers. After the third bout, the blood lactate concentrations in young wrestlers were between 10 and 11 mmol/L, but in senior wrestlers, the blood lactate concentrations were from 11 to 14 mmol/L ( Karnincic et al., 2009 ; Yoon, 2002 ) or from 15 to 20 mmol/L ( Barbas et al., 2011 ; Kraemer et al., 2001 ). These differences are not age related. They are most likely due to the long training experience and higher training intensity in senior wrestling which causes a variety of metabolic adaptations ( Daussin et al., 2008 ; Friedlander et al., 1997 ; Hashimoto and Brooks, 2008 ). The main difference between the high- and low-quality wrestlers is that the blood lactate concentration in high-quality wrestlers rose statistically significantly for two bouts, while in the low-quality wrestlers, lactate rose only in the first bout ( Karnincic et al., 2009 ). High-quality wrestlers have better fitness levels as a result of more intense training. Metabolic adaptations induced by training allow high-quality wrestlers to work longer and under higher loads. They also produce more blood lactate at higher intensity levels and have faster lactate clearance. This may also be influenced by greater buffering capacity, what allows well trained athletes to produce ATP through glycolysis. The blood lactate concentration in all of the groups in this study rose significantly in the first and second bouts. Therefore, we can assume that the participants are a well-trained group.

To ensure that all of the wrestlers experienced the same recovery conditions, passive rest was chosen because active recovery depends on the intensity of activity ( Menzies, 2010 ). After 5 min of rest, the heart rates decreased to approximately 120 bpm, the glucose concentration continued to rise significantly to 8.5 mmol/L, and the lactate concentration decreased significantly to 8.8 mmol/L from 11.1 mmol/L. Although, it is known that peak lactate values appear approximately 3–8 min post-exercise ( Goodwin et al., 2007 ) and that active rather than passive recovery is more effective at clearing accumulated lactate ( Menzies, 2010 ), all three groups had recovered significantly after 5 min of passive rest. This fact indicates that for participants from a group of high-quality wrestlers, recovery began relatively quickly. The fact that glucose uptake is relatively slow in the first five minutes may be because muscle anaerobic glycolysis depends on muscle glycogen rather than on blood glucose ( Palleschi et al., 1990 ). Well-trained subjects have a higher content of muscle glycogen; thus, the need for glucose uptake may occur later, which may explain why the glucose concentration rises in well-trained athletes. Hyperglycemia in trained subjects is a result of lower expenditures of glucose and not a result of increased glucose production ( Coggan et al., 1993 ). To better explain lactate and glucose dynamics in sport diagnostics, further research into different qualities of youth wrestlers should confirm the hypothesis of this study.

## Conclusion

The research results confirmed that there are no significant differences between the measured concentrations of lactate and glucose before, during, and after wrestling matches between lightweight, middleweight, and heavyweight young wrestlers. The only difference between weight categories is the relatively low correlation between lactate and glucose for lightweight wrestlers after the third bout and after 5 min of recovery. However, this difference did not affect the lactate or glucose dynamics.

## Practical applications

The study evaluated the blood lactate dynamics for youth wrestlers (before, during, and after the fight) (Fisher LSD 1 < 2 < 3 = 4 > 5) and the glucose dynamics (Fisher LSD 1 = 2 < 3 < 4 < 5) for all weight classes. A better understanding of lactate and glucose metabolism during wrestling matches is important for wrestling coaches. The fact that body weight does not affect lactate and glucose dynamics during wrestling matches, during which coaches need to evaluate a wrestler’s anaerobic energy status, is also very important.

## Figures and Tables

**Figure 1:**
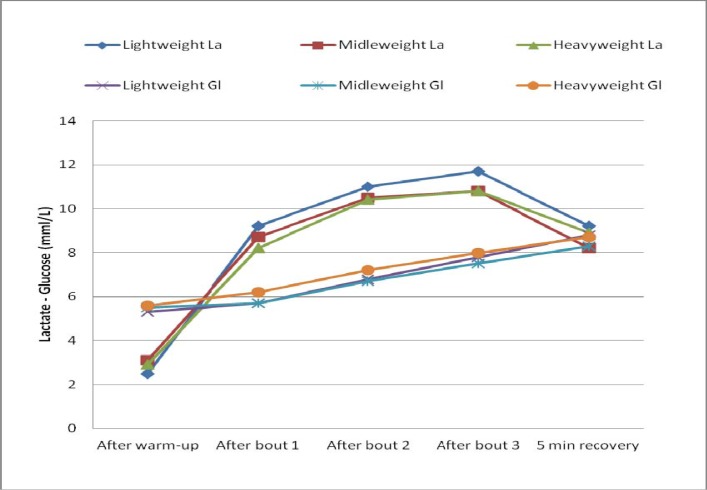
*
Lactate and glucose curves in the Greco-Roman wrestling match There is no difference in blood lactate and glucose concentration between the groups (Fisher LSD test).
*

**Table 1:** *
Physical characteristics, training experience and national ranking of analyzed wrestlers
*

	Lightweight (n 20)		Middleweight (n 20)		Heavyweight (n 20)	
	mean ± SD	min / max	mean ± SD	min / max	mean ± SD	min / max
Age (yrs)	16.4 ± 1.8	15.0/20.0	16.6 ± 1.0	15.0/18.0	17.4 ± 1.8	15.0/20.0
Experience (yrs)	5.7 ± 2.5	1.0/10.0	4.3 ± 2.0	1.0/8.0	5.8 ± 3.4	2.0/12.0
Body mass (kg)	57.4 ± 6.3	45.0/66.0	69.9 ± 1.7	66.0/73.0	88.0 ± 13.0	73.0/120.0
Body height (cm)	168 ± 8	150/183	176 ± 4	168/187	180 ± 6	170/193
BMI (kg/m ^ 2 ^ )	20.3 ± 1.7	17.2/23.6	22.6 ± 1.2	19.7/25.2	26.8 ± 2.8	21.1/32.2
Ranking	3.4 ± 2.6	1.0/10.0	6.4 ± 2.2	1.0/9.0	4.2 ± 3.2	1.0/9.0

**Table 2:** *
Lactate, glucose and heart rate profiles (mean ± SD) of wrestlers. Differences inside the groups (Fisher LSD test) and correlation significance between blood lactate and glucose for each group
*

	La - mmol/L	Gl - mmol/L	HR - bpm
LIGHTWEIGHT (n=20)			
Warm-up	2.5 ± 0.6	5.3 ± 1.1	104.2 ± 11.0
bout 1	9.2 ± 2.3 *	5.7 ± 0.9 *	189.7 ± 9.6
bout 2	11.0 ± 3.0 *	6.8 ± 0.9 *	189.4 ± 7.7
bout 3	11.7 ± 3.1	7.8 ± 1.3 * ^¥^	190.6 ± 9.1
5 min recovery	9.2 ± 3.2 ^†^	8.8 ± 2.2 * ^¥^	120.1 ± 7.6

MIDDLEWEIGHT (n=20)			
Warm-up	3.1 ± 0.8	5.5 ± 0.8	106.6 ± 15.0
bout 1	8.7 ± 2.4 *	5.7 ± 1.0 *	183.9 ± 8.7
bout 2	10.5 ± 2.0 *	6.7 ± 1.1 *	188.1 ± 5.7
bout 3	10.8 ± 2.5	7.5 ± 1.1 *	188.5 ± 7.4
5 min recovery	8.2 ± 2.9 ^†^	8.3 ± 1.7 *	122.5 ± 6.6

HEAVYWEIGHT (n=20)			
Warm-up	2.9 ± 0.7	5.6 ± 1.3	106.6 ± 12.7
bout 1	8.2 ± 1.9 *	6.2 ± 1.6 *	188.8 ± 5.8
bout 2	10.4 ± 2.2 *	7.2 ± 1.6 *	191.0 ± 8.0
bout 3	10.8 ± 2.1	8.0 ± 1.4 *	189.2 ± 8.2
5 min recovery	8.9 ± 2.7 ^†^	8.7 ± 1.7 *	124.9 ± 8.1

* *significant increase for lactate or glucose at p<0.05,*

† *significant decrease for lactate or glucose at p<0.05.*

¥ *correlation are significant at p<0.05.*
